# Is there no “I” in team? Potential bias in key informant interviews when asking individuals to represent a collective perspective

**DOI:** 10.1371/journal.pone.0261452

**Published:** 2022-01-14

**Authors:** Whitney Fleming, Brittany King, Kerrick Robinson, Eric Wade, Brian Erickson, Jackie Delie, Ricardo de Ycaza, David Trimbach, Ana Spalding, Kelly Biedenweg

**Affiliations:** 1 Department of Fisheries, Wildlife, and Conservation Sciences, Oregon State University, Corvallis, OR, United States of America; 2 Architecture and Town Planning, Technion—Israel Institute of Technology, Haifa, Israel; 3 Department of Natural Resources and Society, College of Natural Resources, University of Idaho, Moscow, ID, United States of America; 4 School of Public Policy, Oregon State University, Corvallis, OR, United States of America; University of Hradec Kralove: Univerzita Hradec Kralove, CZECH REPUBLIC

## Abstract

This paper sought to understand the extent to which, and how individuals use personal or collective language when asked to articulate sense of place from a collective perspective. Understanding a collective sense of place could illuminate place-based connections in natural resource industries, where it is as groups or as institutions that organizations interact with the environment rather than as individuals. While there are well known methods for collecting information about sense of place at the individual level, there is a gap in understanding the best method to collect information at a collective level. We examined the use of key-informant interviews as a method to understand collective sense of place. In Bocas del Toro, Panama, ecotourism and environmentally based organizations are becoming more prolific due to abundant natural resources, making it an interesting case study for understanding sense of place from an organizational perspective. The use of personal and collective language is examined though in-depth semi-structured interviews from 15 environmentally-oriented organizations with a total of 17 interviews. This study specifically examined whether and how key informants, when prompted to speak for their organization, spoke collectively, reflecting a collective perspective versus their own. Methods included both quantitative analysis of personal versus collective language use frequency, and qualitative examinations of how individuals used personal versus collective language. Our results indicated no difference in the frequency with which individuals use personal versus collective language. We found that how individuals situated their perspectives into an organization reflects a complex personal and collective point of view reflecting five themes of personal versus collective language use: 1) sole personal perspective, 2) sole collective perspective, 3) distinction between collective and personal perspective; 4) organization perspective with insertion of “I think”; and 5) personal and collective perspective about organization and greater community. Our research identifies a previously undiscussed potential bias of key informant interviews. These findings have implications for how researchers approach collecting information beyond the individual level.

## Introduction

There is growing interest in understanding how organizations contribute to environmental sustainability through their interactions with the natural environment, including their sense of place [1]. Sense of place studies at an organizational level have implications for environmental sustainability and how the understanding of organizations is framed [2, 3]. Researchers suggest that if individuals working in an organization have greater place-based connections to the land, the organization might make decisions that promote sustainability [4]. However, most sense of place studies focus on the individual as a unit of analysis, while some have examined the environmental sustainability of organizations based on their collective actions. The conceptualization of sense of place for a whole organization is not well understood.

There are different approaches to elicit sense of place, notably quantitative versus qualitative, which vary by the sense of place construct under investigation and the scientific field (i.e., anthropology, geography, psychology) [5, 6]. Semi-structured in-depth interviews are a common method to prompt sense of place [7–9]. Aggregation of individual responses occurs in both quantitative (e.g. [10, 11]) and qualitative studies (e.g. [12, 13]). Alternatively, researchers could recruit several individuals to speak for the larger group or organization. Referred to as key informants in social science research, these individuals can provide researchers an ‘in’ with a community because of their comprehensive knowledge and ability to communicate the community’s culture [14]. In an organization, key informants may be leaders, managers, or elected representatives that can share information about the development, history, and establishment of an organization’s mission and vision [14]. Due to how key informants are situated within an organization, they are valuable resources and could shed light on collective, or organization level, sense of place. As individuals situated within a specific place and community, key informants also have a personal, or individual, sense of place. Hanlon [15] used key informants to understand sense of place in hospitals and found differences in where interviewees situated their organization within a broader or narrower context.

Our study explores the implications of interviewing individual key informants and asking them to represent the collective organization perspective rather than their perspectives. Below, we provide background literature on (1) sense of place and its importance to organizations in natural resources, and (2) understanding the perspective elicited from individuals through language use. We present the background on sense of place to understand its importance to measure; however, this study does not measure sense of place or its implications for the environment at the organizational level. This study does measure individual use of language when describing sense of place related to organizations to understand our objective.

### Research objectives

Understand whether the language used by key informants is representative of the collective or personal experience when interviewed about collective sense of place at an organizational level. Understanding this objective is the first step in determining how to measure sense of place at a collective or organizational level rather than an aggregated individual level. We also wanted to explore the questions:

Does the frequency with which individuals use personal language differ from the frequency with which they use collective language?

What are the ways in which individuals use personal language compared to collective language when asked to speak from the collective perspective?

### Sense of place and organizations

The sense of place concept derives from human geographers who sought to understand the connection and meaning of *place*, a physical setting with an assigned meaning [16–18]. Sense of place has been explored across various academic fields and embodies a multidimensional construct [19]. Founding geographers consider sense of place to be attachment and meanings associated with a specific locale [16]. Psychology researchers use this concept to understand an individual’s cognitive connections to a place [5, 20]. Following Masterson et al.’s [21] and the social-ecological conceptualization literature of sense of place, we use the concept as an umbrella term, comprised of two psychological domains: place meaning and place attachment. Place meanings are descriptions of what a place is, what it conveys, or what it is like [21]. Place attachment is an evaluative emotional bond (positive or negative) between individuals and place, divided into the two subdomains of place identity and place dependence. Place identity is the evaluation of one’s personal identity with a physical setting, and place dependence is a behavioral dimension that captures how a place provides instrumental benefits such as satisfying personal needs or goals [21].

Sense of place is important for people’s connection to the natural environment, including their environmental stewardship [8, 22, 23]. From an organizational level, applying place-based theory can help reframe the traditional understanding of organizations to include the relationship between organizations and nature [24, 25]. Research suggests the importance of organizational perceptions of place and demonstrates how a place can affect organizational processes [2]. Organizations are physical places themselves with individuals who live in these places with ecological and social contexts [2]. Each organization interacts with the natural environment somehow but may not consciously acknowledge how these interactions influence their decision-making processes [4]. These interactions are especially true for specific industries. For example, ecotourism, sense of place in social marketing promoted business, and sustainable tourism development [26]. Sense of place-related papers have focused on an individual’s or a community’s sense of place [1]. By applying this operationalization of sense of place to an organization, researchers may begin to understand collective sense of place. A collective sense of place, at an organizational level, could help understand locally meaningful place connections and environmental actions.

### Individual versus collective perspective and language use

Individuals of an organization go through a process called organizational identification, which is how individuals identify themselves with the organization [27–29]. The more salient an individual’s organizational identification, the more an individual may see themselves reflected in the organization [30, 31]. Strong organizational identification can result in organizational values, goals, and norms becoming salient for an organization [28]. It allows individuals to reflect on the organization’s interest rather than their self-interest [28, 32]. For example, Krienier et al. operationalized boundaries as ‘mental fences’ that individuals create and renegotiate during the process of making sense of who ‘I am’ and who ‘we are’ [33]. The study found individuals of organizations possessing multiple, potentially unstable, organizational perceptions that can result in differing accounts of the organization amongst its members [33]. Others have suggested that language plays a role in forming organizational identification and revealing the strength of identification [30, 34]. An organization that uses collective language can strengthen collective culture [35]. How an individual of an organization uses language, including the use of personal and collective markers (e.g., “I” and “we”), can both strengthen and weaken the representations of a member’s organizational identification [30]. Examining how leaders use collective rather than personal pronouns shows how they situate themselves within an organization [36]. Exploring language use is an essential first step to identifying collective versus personal perspectives.

### Study area

To assess how key informants speak about sense of place—using personal or collective language—we conducted fieldwork in Bocas del Toro, Panama. Our objective in this paper was not the original research intention. We initially sought to understand organizations sense of place. However, we began to question during data collection whether individual interviewees were representing their organization. When in the field, we noted a commonality that respondents may not be speaking collectively for their organization even when prompted. It became clear before finishing all interviews, suggesting data saturation, that some participants used collective language, some used personal, and some used both. Because of this, this paper explores the extent to which individuals speak collectively or personally about sense of place. That said, Bocas del Toro was an ideal study area because of the many organizations, including government, non-profit, and businesses, that interact or rely on the natural environment.

Bocas del Toro is a province of Panama located in the northwest of the country bordering Costa Rica to the west and the Caribbean Sea to the north. A large mainland portion plus a chain of islands off the Caribbean coast boasts a rich environmental and biological diversity, comprised of physically and ecologically interconnected terrestrial and marine habitats [37]. Its population includes Indigenous ethnic groups that coexist with a sizeable Afro-Antillean community and other Panamanian mestizos. Bocas del Toro is also home to many foreigners, attracted by the region’s natural environment, economic opportunities, and low living costs [37, 38]. Tourism is the major economic activity for insular Bocas, which has become one of Panama’s main tourist attractions. Bocas has seen a rise in ecotourism initiatives, businesses, and economic activities in the last few decades [39]. It also houses various local and foreign organizations dedicated to research, education, conservation, and management of natural resources [40].

Isla Colón, the main island, houses the regional capital city of ‘Bocas Town,’ a small, vibrant town and focal point for social and economic activities. The influx of foreigners and foreign businesses into the region has displaced and marginalized locals, spurred high social and economic inequalities, and generated tensions and competition between the locals and foreigners [37]. Similarly, these activities have significantly impacted the region’s natural environments and caused land tenure conflicts [41]. These dynamics are ingrained in the region’s colonial history [42], producing a deep-rooted sense of place fueled by a feeling of ownership from locals and jealousy towards foreigners. These characteristics of Bocas del Toro, its people, and the various organizations in the region make this an ideal place to study the role of place, including place dependence and place meaning, for organizations.

## Methods

### Data collection

The Oregon State University Institutional Review Board declared this study was research not involving human subjects because the questions asked were about the organization and not about the individual. As stated in the methods section, consent to participate was either given verbally or via written communication, depending on the participant. In the case of verbal consent, participants were given information at the start of the interview, and then asked if they were okay being recorded and if they wanted to proceed. No minors were interviewed. No personal information about participants was collected or retained.

We used purposive sampling to identify organizations for this study with a combination criterion and convenience based purposeful strategy [43]. The criteria for our sampling included 1) the organizations should be in business, non-profit, or government sectors, 2) the organization should be located and conduct its primary business in the study location (Bocas del Toro), and 3) that the mission of the organization should be directly related to the natural environment. Based on organization’s websites, text documents to determine mission statement suitability, and two of the authors’ prior research experience in the region, we contacted a total of 15 organizations via email to participate in the study. All organizations agreed to participate and nominated one or more individuals to be interviewed based on their role within the organization and availability. Nominated individuals’ role and position in the organization were critical for selection, namely that they were key informants, individuals who generally are considered to have more extensive knowledge of groups and data can be obtained in a shorter period of time [44]. Each interviewee agreed either verbally or in electronic communications to participate. To facilitate the multilingual nature of Bocas, respondents selected their preference for interviews in Spanish or English (please see S1 File). Three of the study authors who conducted interviews are originally from Panama, while four speak Spanish fluently, and three more have an intermediate level of Spanish. All authors but one author are fluent in English. At the time of the study, three authors had PhDs, two with positions as Assistant Professors and one with a position as a postdoctoral scholar. All three have extensive backgrounds in conducting qualitative studies using in-depth interviews, one with years of prior research in the study location. The remaining authors were graduate students at the time of the study. All authors participated in conducting interviews where more junior researchers were supervised by more senior researchers. The graduate students spent one quarter in a dedicated class preparing to conduct the study. Half of the authors are female, and half are male.

In December 2018, we conducted in-depth semi-structured interviews with government (*n* = 1), non-profit (*n* = 5), and business (*n* = 11) organizations (S2 File). One business opted to have three individuals interviewed, bringing the total interview count to 17 individuals. While the intent was to have one key informant for each organization, the authors did not see harm in conducting the interviews with the two additional participants as it had potential for interesting comparisons. Interviews were conducted on-site at each organization in a space chosen by the interviewee (office or similar) with only the participants and the researchers present. Interviewees were informed on the goals of the study and asked if they had any questions at the end of the interview. No relationship beyond the scope of the study was established with participants. We audio-recorded and transcribed all interviews, which lasted anywhere between 15 minutes and one hour. Transcripts in Spanish were translated to English using Google Translate and checked for accuracy by a co-author from Panama fluent in Spanish and English. Interviews consisted of three parts; (a) a cognitive mapping activity about organizational place meaning, (b) agreement statements asking about organizational place dependence where individuals were asked to elaborate on their (dis)agreement, (c) and demographic information for the organization.

### Data analysis

The data we analyzed are narrative data from the interview recordings that people shared as they engaged in the interview with two main tasks. A cognitive mapping exercise asked them to identify and sort cards associated how they would describe Bocas as important to them. The specific method used in the interview was Conceptual Content Cognitive Mapping (3CM). 3CM is a method used to elicit perceptions for complex domains [45] where participants are asked to spatially and visually represent their understanding of a topic with verbal explanation. In the 3CM method, we gave participants 26 concept cards, in Spanish or English, and asked: *please select the concepts that best describe why Bocas is important to your organization*. Interviewers would remind individuals to speak for their organization and to explain their choices. After participants choose their concept cards, they were asked to rank the concepts by perceived importance to their organization and asked to describe their reasoning for concept selection and ranking. Following 3CM, we presented participants with four questions to understand place attachment. Participants were asked on a 4-point agreement scale if their organization would agree (1) or disagree (4) to the following four statements: (1) We could do our work outside of Bocas Del Toro; (2) Bocas Del Toro is the best place to do our work; (3) Our work is necessary in Bocas Del Toro; (4) Over time, Bocas has become more important for our work. Participants were asked to elaborate on their choice and interviewers also asked follow-up questions based on responses.

To understand the use of collective and personal language we used a combination of discourse and content analysis. We uploaded interview transcripts into NVivo, a qualitative data analysis software, and ran a query to count the number of times individuals used personal language -the words “I,” “my,” and “me”. The total number of instances where these three words used were summed for total personal language use per interview participant. We ran the same analysis for total collective language used with -the words “us,” “we,” and “our”. Text from the interviewer was removed before running these queries. The percentage of time an individual used personal versus collective language was calculated for each participant using the total number of instances for each language type. Using a Wilcox Signed Rank test, we calculated whether there was a significant difference between an individual’s frequency of use of personal and collective language. To further understand the meaning behind these collective and personal statements, we coded interview transcripts. All interviews were coded by two co-authors using qualitative coding for personal and collective language and intercoder reliability was assessed using Cohen’s kappa. We created only two codes: “personal” and “collective” based on the use of the aforementioned keywords (I, me, my; we, us, our). Segments and passages where these words were used were coded with either or both codes and qualitatively explored for context and content. We identified co-occurrences where participants use language coded as personal (e.g., for me, I think) while also using language coded as collective (e.g., we, our) to speak about the organization. Themes in personal versus collective language use were derived from the data.

## Results

On average participants used personal language (I, me, my) 51% of the time and used collective language (we, us, our) 49% of the time (Table 1). There was no significant difference between the overall frequency of use of personal versus collective coded language (*p* = 0.851). Differences in personal and collective language used by organization type were not calculated due to our small sample sizes.

**Table 1:** The use of personal and collective language by key informants in interview responses.

	Personal^a^ (I, Me, My)	Collective^a^ (We, Us, Our)
Business (Cooperatives, Community-based Tourism)		
Organization 1	13	87
Organization 2	50	50
Organization 3 (1)	24	76
Organization 3 (2)	79	21
Organization 3 (3)	89	11
Organization 4	17	83
Organization 5	50	50
Organization 6	0	100
Organization 7	100	0
Organization 8	23	77
Organization 9	87	13
Mean (*M*)	48	51
Non-Profit (Conservation, Education & Research)		
Organization 10	72	28
Organization 11	71	29
Organization 12	37	63
Organization 13	50	50
Organization 14	48	52
Mean (*M*)	56	44
Government (Tourism)		
Organization 15	55	45
Mean (*M*)	55	45
Overall Mean (*M*)	51	49

^a^Cell entries are percentages (%) personal (I, Me, My) and collective (We, Us, Our) language used by key informants in their responses to place meaning and place dependence questions.

From coded sections of personal and collective language use, we identified five broad categories of language used, or major themes, in the interviews. The first two types were 1) the sole use of personal language, typically including “I” and “me,” and 2) the sole use of collective language such as “we” and “our” to speak on behalf of the organization. The sole use of personal language included statements such as:

*“I don’t like to use the word beautiful*. *Beautiful means feeling*. *I’m trying to be a little bit more practical*, *you know*?*”* (Nonprofit)

In these instances, the respondents did not use any type of collective language and made no reference to the larger organization or group. Instead, they only used personal language to describe their own perspective and thoughts.

Where the sole use of collective language included statements like:

*“…we as an organization have developed some environmental investment projects*, *we take care of nature*, *we take care of marine biodiversity*, *because without fish*, *there are no fishermen*.*”* (Business 1)

In these instances, the respondent explicitly referenced the organization in their responses or used collective language to represent the organization or group. In segments such as this, the interviewee did not use personal language or a reference to the individual at all, rather spoke only using collective language words.

The other three major themes of language use emerged from the co-occurrence of language. Coding for personal and collective language revealed a total of 205 personal segments and 217 collective segments, 86 of which overlapped (25.6% co-occurrence, Kappa = .79). From this overlap we identified the other three categories of language used: 3) distinction between collective and personal perspective; 4) organization perspective with insertion of “I think”; and 5) personal and collective perspective about organization and greater community.

The third theme revolved around the use of a clear distinction in a single thought between the individual and their organization. This occurred when individuals situated their specific role, perspective, or experience within the greater organization in Bocas. Such as this individual from a nonprofit:

“*another thing we do in Panama is one of the things because, and even related to garbage, because [the organization] started in Panama with beach cleanups*. *You know?…I think that here I know, I am not so focused in these cleanups. Sometimes I do it*.”

In this instance, the interviewee describes the role of the organization in beach clean-ups, but clarifies the distinction between the organizations role in clean-ups and their own role in clean-ups.

Or as this individual from a different nonprofit:

“…*ours, at the moment, since we’re still starting out, probably have more of an effect on the students versus the community at this moment*. *Since this is only my first programs that I’m running, I’m trying to get it to be more involved in the community*…”

Again, this participant describes an effect the organization has using the collective words “ours” and “we’re” but adds their personal effect subsequently as see through the use of personal words such as “my” and “I’m.” In these passages, individuals used collective language to talk about the organization or group, but separated themselves or more specifically defined their individual role.

The fourth theme, organizational perspective, showed it was common for an individual to say “I think”, “I believe”, or something of the sort, that was a quick interjection of personal language in an otherwise collective way of speaking. For example, an individual working at a business stated:

*“Because what we are looking for is that other organizations can also develop as we do*, *not that we feel ownership of where we come from*. *I think that is the idea*.”

Here, the majority of the segment is expressed from a collective perspective, but the individual adds a quick interjection of personal language.

Last, we identified instances where individuals used collective and personal language to speak about greater individual, organizational, or community identities in and around Bocas. There was an intermixing where the line blurred between the personal and collective perspective and its connection to organizational and community. Such as with this individual from a business:

“*This is a small island, but we have people from mainland*…, *from David [City], from where they are coming from. Opportunities are economic for the family, poor family, this is very good*. *That is what I am going to choose*.”

Or this individual from a nonprofit:

“*For me the opportunity for education is important because here there is a grade level where we can no longer study more types of branches of study, quality of study or education*. *Here in La Colón it only counts up to the sixth year. If you already got there, for us to continue studying we have to go to other provinces that have a much better education. So, for us it is difficult*.”

In these passages, individuals may speak about their organization’s sense of place and connection to the environment, but also reference larger issues related to the environment or community and how they fit within that context. In these instances, an individual does not define a specific role they have, or clarify it is their opinion as in themes three and four, but instead more wholly mix their own identity into the collective perspective.

## Discussion

With increasing interest to understand the connections between organizations and the natural environment, sense of place-related studies can provide insight into these relationships. Sense of place has important connections to environmental behaviors and actions, yet is generally measured at an individual level [46]. Our study wanted to understand a broader collective sense of place, at the organizational level, to understand potential implications for organizational environmental behaviors by using key informants to represent their organization. We found an interweaving of individual and collective narratives that made measuring sense of place from a collective or organizational level difficult from an individual; thus, implying difficulty for researchers in measuring collective sense of place in this way.

We did not find a statistically significant difference between the frequency of personal and collective language used by respondents in Bocas del Toro. Our results showed that respondents used personal language, just as often as they used collective language in response to sense of place questions about their organizations. Because one organization opted to have three key informants interviewed, this showed that personal and organizational perspectives differed between participants from the same organization. Asking participants to speak about their organization to provide a collective representation of sense of place at the organizational level, we expected the use of collective language to be more frequent. Using personal language, however, does not automatically suggest participants’ inability to speak for the organization. Depending on the use of personal language, sense of place information obtained from our study’s respondents may represent their organization. Therefore, it was important for us to determine how participants used personal and collective language and their interpretation of sense of place.

Our results identified the overlap of organizational and personal identification through the lens of language co-occurrence. Instances where respondents mixed personal and organization perspectives occurred when participants described aspects of their organization or its work, but pulled themselves out of that narrative to describe their role. This may be an example of the permeability of boundary dynamics [33] where individuals internally negotiate the boundaries between their individual perspective and the organization’s by speaking on their individual perspectives about the organization. Organizational identification, is a reflection of a member’s sense of identity and how they define themselves as similar to the organization [27, 29]. A weak level of organizational identification, and therefore, identity suggests that asking a single key informant to speak on behalf of an organization, without first assessing their level organizational identification may not be the appropriate approach to measure sense of place at a organizational level.

Alternatively, participant’s choice of language (I think) may reflect their interaction with the interviewer. Scholars refer to “think” as a cognitive verb which, when used with a first-person (“I”), may be viewed as a parenthetical expression of commitment to a subject [47, 48]. Researchers have suggested that using the parenthetical, “*I think*,*”* the speaker invites the hearer to adopt their perspective towards a particular claim [47, 49]. This indicates a type of self-referencing where individuals use both personal and collective language to situate themselves within the group [35]. However, when used frequently by participants, we found it unclear whether a respondent was speaking from the organizational perspective or their own. There were also several instances of overlap of personal and collective language when respondents spoke about their perspective on the Bocas Del Toro Region or their immediate community. In these statements, interviewees used “I” in personal references, whereas “we” referred to the community or region as a whole. These statements shed light on the importance of the region to the participants and their community, suggesting that these statements may represent sense of place at an individual, rather than collective level. Overall, we found that measuring collective sense of place from a key informant is problematic.

Our study is not free from bias and limitations. The infrequent collective language use may reflect cultural aspects of Bocas del Toro or differences in spoken language (Spanish versus English). Due to the focus of this study, one important consideration is that more than half of the interviews were originally conducted in Spanish and required translation. We determined the translation to have face validity. While cross-language research is not uncommon, researchers have identified some of the methodological challenges of conducting these studies. One challenge is ensuring “conceptual equivalence,” which focuses on retaining the meaning and context of the responses across languages [50, 51]. If translation is not done in a careful and meaningful way, research data and context can become lost in translation and impact data analysis [50]. This study involved a quantitative and qualitative analysis heavily focused on the text as it was presented in English. While we believe we took the appropriate step to translate the interview, our qualitative analysis may lack important cultural context about the participants and their use of language. Further research would benefit from understanding the cultural differences in language use in studies of identity and sense of place in organizations.

The goal of this study was to explore whether the language used by key informants represented the collective or individual experience for environmental organizations in Bocas. As pointed out by Lokot [52], key informant interviews are considered a reliable and ubiquitous part of qualitative research, but as she pointed out, potentially problematic because of potential bias due around who key informants are representing. Our research identifies a previously undiscussed potential bias of key informant interviews. We hypothesized that participants possessed a collective sense of place and that this would be reflected in their responses. Our findings reveal that collective language use varied across participants and interview responses. While some participants were able to distance their personal perspective from the organization, others mixed perspectives so frequently, disentangling them was impractical. Based on the complexity of personal and collective perspectives elicited from participants, our findings suggest using a key informant as the sole source to elicit sense of place from a collective perspective is inadequate. It does, however, have interesting implications for what a collective sense of place might mean. Future researchers may consider the collective sense of place concept when measuring sense of place and how an individual’s personal and collective experience adds to or hinders environmental sustainability goals. Additionally, further research in other locations could test individual and collective perspectives to identify similar or dissimilar results.

## Conclusion

This study sought to measure a collective sense of place and relay findings back to environmentally centered organizations in Bocas del Toro, Panama. Instead, we learned a practical lesson about the extent that individuals spoke for their organization and whether interviewing a single member of an organization is a viable means of collecting data about a collective sense of place. Our results indicated that while participants could provide collective representations of the organization’s perspective, they also showed the tendency to speak from personal perspectives. Our results revealed the importance of collective versus personal perspective missing from our original study. As seen in our results, the boundary between the two can be permeable, with each perspective having the potential to seep into the other. In our case, individual identities presented themselves in our attempts to collect data at an organizational level, highlighting a potential issue in using a sole key informant to represent an organization. Krienier et al. [33] discusses identity boundary dynamics, “to oversimplify either the individual’s or the organization’s identity as monolithic ‘boxes’ would, in most cases, produce grave misconceptions of both.” While studies have highlighted the potential values of incorporating sense of place into organizational research [2] our study demonstrated that emphasis should also be placed on disentangling collective and personal perspectives and how these constantly shifting measures influence, and are influenced by, the places in which these organizations call home.

## Supporting information

S1 File Interview guide in English and Spanish. This is the interview guide used in this study provided in English.
(PDF)

S2 File Original transcripts. This zip file contains the original transcriptions of the interviews used in for this study.
(ZIP)
