# Supplementary material for: Is there no “I” in team? Potential bias in key informant interviews when asking individuals to represent a collective perspective
Source: PLoS One. 2022 Jan 14;17(1):e0261452. doi: 10.1371/journal.pone.0261452 (PMC8759660; doi:10.1371/journal.pone.0261452)
Supplement: S1 File — This is the interview guide used in this study provided in English. (PDF) [file pone.0261452.s001.pdf]

Appendix 1

Interview Guide – English

Good [morning/afternoon/evening] Ma'am/Sir; How are you doing today?

My name is XXX, we are students at OSU. We are interested in understanding the experiences of your organizations and how/whether your organization is dependent on Bocas, including people, cultures, environment, etc. We plan to use the information to compare with other organizations and prepare a report. If you are interested, we are happy to provide you a copy of the final results. Depending on how much you want to share, the process should last approximately 45 minutes. Do you have any questions before we move on? If you have any questions at any point, you can stop the process and let me know.

Part I – Cognitive Mapping Exercise

From these cards, please select the ones that best describe why Bocas is important to your organization. If there is not a concept or word here that you would like to add, please feel free to do so using these empty cards. Now that you've selected your cards, can you rank those cards in order of importance to your organization? *(Remind participants they are speaking for the organization)*

Part II – Logistic Information

1. What is the purpose of your organization?

2. What is the structure of your organization and what is your position within this structure?
3. Please describe some of the main activities of your organization.
4. How long has your organization been in Bocas Del Toro?
5. Where is your organization based?
6. How many employees are in your organization?
7. Which category/categories best describe(s) your organization?
    1. Government
    2. Research and Education
    3. Community-based
    4. Conservation
    5. Business

Part III – Sense of Place Questions

Indicate how much your organization would agree or disagree with the following statements, following your answer and response please explain:

1. Strongly disagree
2. Disagree
3. Agree
4. Strongly Agree

DK- Don't Know

1. We could do our work outside of Bocas Del Toro.

2. Bocas Del Toro is the best place to do our work.

If 3 or 4, what other organizations like yours would answer the same?

(Open-ended)

If 1 or 2, where else would you be able to do your work?

3. Our work is necessary in Bocas Del Toro.

4. Over time, Bocas has become more important for our work.

* Thank you for your time. Do you have any questions for us?

(take notes of questions)

* We'd like to invite you to a group meeting on Friday at 11:30am at the ATP office to share and discuss our findings from this week. The discussion will be followed by lunch at 1pm. Would you like to join us?

Interview Guide – Spanish

Buenos(nas) [días/tarde/noche] señora/señor; ¿cómo está usted hoy?

Mi nombre es XXX, somos estudiantes de OSU. Estamos interesados en conocer sobre las experiencias de su organización y su relación de dependencia con Bocas, incluyendo a su gente, su cultura, el ambiente, etc. El propósito principal es de utilizar esta información para comparar las diferentes organizaciones y preparar un informe con los resultados. Si le interesa, con mucho gusto le podemos compartir una copia de los resultados. Dependiendo de cuanto usted quiera compartir con nosotros, esta actividad no debe tomar más de 45 minutos. ¿Tiene alguna pregunta antes de que continuemos? Si tiene alguna pregunta en cualquier momento me lo hace saber y paramos el proceso para aclarar sus dudas antes de continuar.

Parte I – Mapa Cognitivo

De las cartas que le hemos entregado, por favor selecciones las que más describan la importancia de Bocas para su organización. Si hay un concepto o palabra que usted quisiera agrega, siéntase libre de hacerlo utilizando una de las cartas en blanco. Una vez haya hecho su selección, por favor ordene las cartas en base al orden de importancia que cada uno de estos conceptos/palabras representa para su organización. (Recordar a los participantes que están hablando en representación a su organización).

Parte II – Demográfico

1. ¿Cuál es el propósito principal de su organización?
2. ¿Cuál es la estructura de su organización y cuál es su posición dentro de esa estructura?
3. Por favor describa las actividades principales que desempeña su organización.
4. ¿Cuanto tiempo lleva su organización en Bocas Del Toro?
5. ¿En dónde está ubicada la sede principal de su organización?
6. ¿Cuántas personas trabajan en su organización?
7. De las siguientes categorías, indique las que mejor describan a su organización:
    1. Gobierno
    2. Educación e Investigación
    3. Organización de Base Comunitaria
    4. Conservación
    5. Negocios

Parte III – Entrevista Semi-estructurada

Indique si su organización está de acuerdo o en desacuerdo con los siguientes comentarios, y posterior a su respuesta por favor explique.

1. Muy en desacuerdo
2. desacuerdo
3. De acuerdo
4. Muy de acuerdo
5. NS – No sabe

1. ¿Su organización podría realizar su trabajo fuera de Bocas?
2. ¿Bocas del toro es el sitio más adecuado para el trabajo que realiza su organización?
3. Su trabajo es necesario en Bocas
4. A lo largo del tiempo, la importancia de Bocas para nuestro trabajo ha incrementado.

Si la respuesta es 3 o 4 - ¿Qué otras organizaciones responderían a esta pregunta de la misma manera? (pregunta abierta)
Si la respuesta es 1 o 2 - ¿En dónde más podría su organización realizar su trabajo?

* Gracias por su tiempo. ?Tiene alguna pregunta para nosotros?
(anotar las preguntas)

* Queremos invitarle a un taller el viernes para presentar y discutir los resultados de nuestro trabajo durante esta semana. Es a las 11:30am en las oficinas del ATP. Tambien les invitamos a un almuerzo despues. Ud. puede atender?
