# Supplementary material for: Is there no “I” in team? Potential bias in key informant interviews when asking individuals to represent a collective perspective
Source: PLoS One. 2022 Jan 14;17(1):e0261452. doi: 10.1371/journal.pone.0261452 (PMC8759660; doi:10.1371/journal.pone.0261452)
Supplement: S2 File — This zip file contains the original transcriptions of the interviews used in for this study. (ZIP) [file pone.0261452.s002.zip › Agreement Transcripts/CON_Starfish_I(agreement statements responses).docx]

Speaker 2: Promar is a national organization.

Speaker 2: Can work in any parts of Panama.

Speaker 2: Right now would be Bocas but I can work also in other parts of Panama if I needed, okay?

Speaker 2: I also, Promar, we're part of the national organization that is held in the border, a sister, an organization that is together with Costa Rica. I am part of this cycle of community tourism..

Speaker 2: I am part Promar-based. Promar, no, as the other level, that is Alliance a Bocas. But I am in Alliance Bocas, Promar. So it's the same.

Speaker 2: No, I can do it.

Speaker 2: Yeah, anything outside of Bocas.

Speaker 2: I already am.

Speaker 2: I agree, of course.

Speaker 2: We have another organization only that is working conservation that is conservancy, okay?

Speaker 2: The only one and we work together on many things and also I can do the same. The only difference that they have a salary, depending on the organization in the states, the United States or local, but it's not local at the same time.

Speaker 2: Not Panamanian, you know?

Speaker 2: But they can more or less our goals ...

Speaker 2: Very difficult to tell.

Speaker 2: I don't know. Really I don't know. Because for me, necessary, but I don't know if many other perceive the same.

Speaker 2: If I live from Bocas Del Toro, I don't know. I hope that there are other people, because I am Panamanian, no? So many probably I don't know, there may be other people trying to do the same, no? I hope. But I don't know.

Speaker 2: I think so, I think so because in the very beginning, we work ... another thing we do in Panama is one of the things because, and even related to garbage, because Promar started in Panama with beach cleanups. You know? Because in 1991 was the first beach cleanup in Panama and we did it, okay? Panama City. Now it was in the [inaudible 00:33:41] Panama.

Speaker 2: I think that here I know, I am not so focused in these cleanups. Sometimes I do it. I don't know really with cleanups. Now I come back and I lead ... but ... ask me the ...

Speaker 2: Yeah. What was the question?

Speaker 2: Yes, yes.

Speaker 2: We do many other things. Many other things here and ... I remember now where I was going, where I was going. We started with beach cleanups, and here we do, only make, only us, and we do this. We try to solve the problem of the garbage. Not only beach cleanups, you know?

Speaker 2: This is the way, the thing, because I seemed that we are going ahead with the original task, okay?

Speaker 2: Because we are trying to solve the problem of the garbage, not only cleaning the garbage, okay?

Speaker 2: I strongly agree.

Speaker 2: Yeah.

Speaker 2: Yeah.
