# Supplementary material for: Is there no “I” in team? Potential bias in key informant interviews when asking individuals to represent a collective perspective
Source: PLoS One. 2022 Jan 14;17(1):e0261452. doi: 10.1371/journal.pone.0261452 (PMC8759660; doi:10.1371/journal.pone.0261452)
Supplement: S2 File — This zip file contains the original transcriptions of the interviews used in for this study. (ZIP) [file pone.0261452.s002.zip › Agreement Transcripts/CBT_Cow(2)_Translation(agreement statements responses).docx]

**Interviewee 1:** I would say no.

**Interviewee 1:** Disagreement.

**Interviewee 1:** Because supposedly all of us who are organized here, we know what the work area is. We're going to put, I'm going to Chiriqui and I do not know the area, I can not work. However, here, where I move, I can work.

**Interviewee 1:** It is correct.

**Interviewee 1:** Agree.

**Interviewed 1:** Because actually here, if I tell you in a way, not me will understand, but here everything is done, sold, bring all the product is sold, Boca El Toro.

**Interviewee 1:** It is correct.

**Interviewee 1:** Agree.

**Interviewee 1:** Because if I do not bring fish, people do not eat fish.

[laughs]

**Interviewee 1:** I would say no.

**Interviewee 1:** Disagreement. I explain why, because before we had many fishermen. Al Boca El Toro emerge, there is a lot of tourism, and everyone has dedicated themselves to what is tourism, they no longer fish.

**Interviewee 1:** I say all the organizations that are fishermen.
