# Supplementary material for: Is there no “I” in team? Potential bias in key informant interviews when asking individuals to represent a collective perspective
Source: PLoS One. 2022 Jan 14;17(1):e0261452. doi: 10.1371/journal.pone.0261452 (PMC8759660; doi:10.1371/journal.pone.0261452)
Supplement: S2 File — This zip file contains the original transcriptions of the interviews used in for this study. (ZIP) [file pone.0261452.s002.zip › Agreement Transcripts/EAR_Coral_I(agreement statements responses).docx]

Speaker 2: That's a really complicated question. Somewhat agree.

Speaker 2: No, sorry. I would have to say sort of disagree.

Speaker 2: Yeah.

Speaker 2: Yeah, because well ... So-

Speaker 2: I mean, our work here is very much tied to the community and this community is unique unto any other community, so for that reason I can't do my work anywhere else, right? But I can do my work in other places and we can do our work in other places because our focus is on conservation. So yes, we can do the educational aspects. As a marine biologist myself, I need the ocean. So yeah, I could technically work in other places but I'd have to have a whole different set of people. My work is unique in the sense that this community makes it its own unique job.

Speaker 2: It's a tricky question. I don't know.

Speaker 2: Okay. I'm not quite sure how to answer that. I would say I would slightly disagree that I can do this job anywhere.

Speaker 2: Because it is community based, and even though it's research and education, it's highly dependent upon the community and the people here for this particular work.

Speaker 2: Yeah.

Speaker 2: Oh yeah.

Speaker 2: Strongly agree.

Speaker 2: Well, I think because Bocas is ... it's place-based learning, right? Bocas is this place that is experiencing an extreme increase in tourism without the proper supports in place to be able to support it, and being here not only to support the community, but also to observe and record and document what changes are taking place is really important.

Speaker 2: I think certainly Smithsonian. I mean, where else are you gonna go except for maybe the Great Barrier Reef or Belize and just walk out your door and go to a reef, it's like ... Yeah. Maybe in Roatan in Honduras or something like that. But yeah.

Speaker 2: Absolutely. I agree.

Speaker 2: I don't think there's anyone else doing what we're doing. I mean, I-TECH definitely does research and education, but mostly people come in, take their class, they leave. Our students have a sense of connection and place. Smithsonian, same thing. You get people coming in, they do their research, they benefit, they leave. They're not necessarily giving back to the community. I think our students give back to this community and we make sure that they do that. We don't just take, we are here, we're permanent, we have that advantage over a lot of other groups that just pass through. They take information but they don't necessarily share it back with the community.

That's a problem. People, I think, are getting a little fatigued by constantly having people come and ask them questions. They get fatigued just by SFS coming with students who are like, "Can you explain?" And they're like, "Uh-huh." So it's a delicate balance and I think we have to be really aware and cautious of how we're interacting with these communities. And to be as transparent as possible.

Speaker 2: Oh yeah, I strongly agree. I think that's because it's constantly evolving. When I first got here I can tell you, I just feel like the longer you're here the more changes you see, the more development you see, changes to laws, changes to policies and no enforcement of this and it's ... the place is constantly changing from one day to the next and you just don't know what you're gonna get one day. I think it is important to continue to be here. Yeah.
