# Supplementary material for: Is there no “I” in team? Potential bias in key informant interviews when asking individuals to represent a collective perspective
Source: PLoS One. 2022 Jan 14;17(1):e0261452. doi: 10.1371/journal.pone.0261452 (PMC8759660; doi:10.1371/journal.pone.0261452)
Supplement: S2 File — This zip file contains the original transcriptions of the interviews used in for this study. (ZIP) [file pone.0261452.s002.zip › Agreement Transcripts/CBT_Cow(3)_Translation (agreement statements responses).docx]

**Marta:** I do not agree, because we are bocatoreños.

**Marta:** Strongly disagree.

**Marta:** Suddenly I tell her that I was president of the Federation. I feel that my work could be done anywhere, but the sinful bocatoreños are special and this is Bocas del Toro.

**Marta:** It is the most appropriate place.

**Marta:** Strongly agree.

**Eddie:** Do others.

**Marta:** Maybe they would not respond the same.

**Marta:** Very good.

**Eddie** Your job.

**Interviewer:** Is your work necessary in Bocas?

**Marta:** Yes.

**Marta:** Strongly agree.

**Whitney:** Very much agree.

**Marta:** Yes, because I am so intense [laughs]. Also, the fishermen here ... I support the sinners of the whole province and I am in other committees.

**Marta:** Yes.

**Marta:** Strongly agree.
