# Supplementary material for: Is there no “I” in team? Potential bias in key informant interviews when asking individuals to represent a collective perspective
Source: PLoS One. 2022 Jan 14;17(1):e0261452. doi: 10.1371/journal.pone.0261452 (PMC8759660; doi:10.1371/journal.pone.0261452)
Supplement: S2 File — This zip file contains the original transcriptions of the interviews used in for this study. (ZIP) [file pone.0261452.s002.zip › Agreement Transcripts/CBT_Cow(1)_Translation(agreement statements responses).docx]

**Interviewee 2:** First, Marta is the president of the National Federation that covers the entire country. She is president here and is president of the federation in Panama, which is part of that, and also participates internationally. The organization about five years ago, also with me and three others, we were an example of Panama to go to Central America from Guatemala, Honduras, El Salvador to give talks and seminars to different organizations to have an idea how the system is organized.

**Interviewee 2:** For example in Chiriquí, there are other organizations. In the Saints. All the provinces have their organizations as soon as they invite us, we go there to project and teach the system how this is handled here as well, but it also has its rules, different from the system here.

**Interviewee 2:** Agree.

**Interviewee 2:** Our extension is here in the province.

**Interpreter:** They teach how you work here. They teach how you work here.

**Interviewee 2:** We teach next province how we work here about all the fisherman, the reef, how the fish eat, how to sell it, and we show them. We go the next province. Yes, we go Costa Rica, Honduras, Nicaragua, for a week, the administration and go Panama**[unintelligible 00:23:45]** I think they learn where we learn that. Then, **[unintelligible 00:23:51]** for them work just like this.

**Interviewee 2:** That's right. Not infrastructures Because what we are looking for is that other organizations can also develop as we do, not that we feel ownership of where we come from. I think that is the idea.

**Interviewee 2:** Bocas del Toro?

**Interviewee 2:** We are now in Almirante, Bocas del Toro, Bocas Isla.

**Interviewee 2:** This site.

**Interviewee 2:** If we talk about Bocas del Toro, it had to involve Chiriqui Grande, Bocas Isla and Almirante.

**Interviewee 2:** There is no other. Why do I say it? Because to get to Bocas you have to get to Almirante. To get to Chiriqui Grande, you have to get to Almirante. This is the collection center where all the people have to arrive.

**Interpreter:** Strongly agree

**Interviewee:** Agree.

**Interviewee:** Strongly agree.

[laughs]

**Interviewee:** Of course I do.

**Interviewee:** Strongly agree.

**Interviewee:** There are also organized fishing extensions that are for the side of Robalo. They always consult first with the headquarters, which is us, the oldest organization in what is fishing. For example, when there are meetings, Marta invites them and we all participate together. Everyone to know how we get ... That in itself, the whole organization we get along.

**Interviewee:** I do not know what the needs would be in terms of this, but I think they would respond the same.

**Interviewee:** The question?

**Interviewee:** Well, comparing it to the first years when we started, there was much more abundance of fish than there is now, but the sale was very low. There was not much money in that, and the prices were very low. It has been seen today-

**Interviewee:** It has been seen today that it has improved.

**Interviewee:** More now.

**Interviewee:** Tourism has grown, the population has grown, we have made ourselves known more about the country of Panama.
