# Supplementary material for: Is there no “I” in team? Potential bias in key informant interviews when asking individuals to represent a collective perspective
Source: PLoS One. 2022 Jan 14;17(1):e0261452. doi: 10.1371/journal.pone.0261452 (PMC8759660; doi:10.1371/journal.pone.0261452)
Supplement: S2 File — This zip file contains the original transcriptions of the interviews used in for this study. (ZIP) [file pone.0261452.s002.zip › Agreement Transcripts/EAR_Frog_I(agreement statements responses).docx]

Speaker 2: Well, yes. We can do our work outside Bocas, that is definitely, I strongly agree.

Speaker 2: Because, you know, we do research. We can do research everywhere, of course. As I told you, we choose this place because the high biodiversity, the position, and the, and we are in the Caribbean area too. If we wanna, to have a full station in the Caribbean. And probably, if we move from Bocas, move to, for example, Cologne where this is the old place, we can find some but not exactly that. We can do it but not as good as we are here, yeah?

Speaker 2: Yes.

Speaker 2: I strongly agree.

Speaker 2: Yes, I feel like I explain already when I tell you, you know, that we are in a place with so many different environment in a small area. Very protect, and something that I didn't mention yet, it's even that we are quote, in a remote area, the tourism activity help us to have things that are not easy to find in other places. We have an international airport, yeah? We have a better infrastructure to access the area, like water taxis, we have buses, we have external activity that help us to have things. And trucks coming in and out with good and services, and so they can help us.

Speaker 2: Wow. Other organization, I don't know. Probably the others like the one that are doing something like we are doing. There is, where's the name? High Tech is one. The other is F, oh, I forgot the name. It's more other group that brings students to, you know, students. It's this way, you know, like-

Speaker 2: [crosstalk 00:41:26] school for field study. Yes. There you go. I don't know if there's any other, I don't know so much like about business organizations too. I don't know.

Speaker 2: Oh my god. This is hard. That's, my answer could be a bit selfish, you know? Or arrogant. It's necessary. I change to important. I don't know if it's necessary like they do not survive, but we produce the best data information about at least in the marine area or in the scientific side, you know, in the entire republic of Panama. There is no other local institution, university or whatever working as our level.

Speaker 2: Yes.

Speaker 2: Yes, of course.

Speaker 2: Yeah, I strongly agree. Especially now, you know, what, now we are more engaged, there are so many scientists, you know? All over the world interested in the topic of Climate Change, you know? And here we have the possibility to because what I said previously about small area and a lot of, we represent, like, a little piece of the Caribbean. Really tiny area, and then if we understand what happen here using these, like a natural lab, very accessible, very easy to get there? We can extrapolate this from the rest of the Caribbean. You know, we have people who are invertebrates, like turtles of low oxygen. [inaudible 00:44:29] nutrients, micro currents, a lot of different little things, you know, and those finding are really comparing with other areas are happen to understand better what happen now in term of the rise of the temperatures? Recently, last week when you visit us we had a micro-marine micro workshop.

This is new topic for me, really, I made the same expression like you are using. You know what they brought to us is like, okay, imagine you are not just in the ocean here and we are everybody in the same condition. If the temperature is rising, everybody feeling that, but we are one organism. You don't imagine if I get you and I put you into the blender, and give that, and they run the DNA I would find you, of course you, here, and I would probably find more thousand and thousand and thousand of other organism that live with you. You know? That is why they want to others to bring up to us, you know what what happened with us is what happened with his thousand and million of microbes that we don't know anything. We are playing, now we are, like discovering or realizing that oh god. We are just one and we have billions here, you know? We need to protection of those guy, because you know what, the destiny of those guy is the same destiny.

You, for example, you eat something, you have million of bacteria in your guts. And if one of them are imbalanced, you could die. Or you can not use in a better way, you know, any kind of system very important for your food you eat. And this is what they are discussing to there are people who expert in coral, in fishes, in blah blah blah, different areas. And they're introducing now the study of the microbes. You know? Yeah, it's super cool, how the interaction, and also, what I really like, I am oceanographer, as an oceanographer I tend to think first of all this is a reef, this is a coral, this is a sea grass, this is whatever. Okay, but everything there is surrounded by the water. This pond here, this one if here, and you have an interaction to explain something that happened here, you can not say this is just using the fish. No, you need to use it because the fish eat the coral or eat the plant or it is eaten by other bigger one, and we try to see that as a big picture.

And if something, if in affect here affect the entire, and you will to know how that system work, you know? This is the only way that later we can say hey, we can modeling for example and say hey if this and this and this is happen, okay, probably we can have this and this and this and this change. You know?
