# Supplementary material for: Is there no “I” in team? Potential bias in key informant interviews when asking individuals to represent a collective perspective
Source: PLoS One. 2022 Jan 14;17(1):e0261452. doi: 10.1371/journal.pone.0261452 (PMC8759660; doi:10.1371/journal.pone.0261452)
Supplement: S2 File — This zip file contains the original transcriptions of the interviews used in for this study. (ZIP) [file pone.0261452.s002.zip › Agreement Transcripts/CBT_Scorpion_Translation((agreement statements responses).docx]

**Emmanuel:** From Bocas del Toro, no.

**Emmanuel:** Disagreement.

**Emmanuel:** Yes.

**Emmanuel:** Yes, okay.

**Emmanuel:** Okay.

**Emmanuel:** There are others such as Nobel Tours. There is another that is Costeños.

**Emmanuel:** Yes, similar.

**Emmanuel:** Yes. I think is.

**Emmanuel:** Yes. Strongly agree.
