# Supplementary material for: Is there no “I” in team? Potential bias in key informant interviews when asking individuals to represent a collective perspective
Source: PLoS One. 2022 Jan 14;17(1):e0261452. doi: 10.1371/journal.pone.0261452 (PMC8759660; doi:10.1371/journal.pone.0261452)
Supplement: S2 File — This zip file contains the original transcriptions of the interviews used in for this study. (ZIP) [file pone.0261452.s002.zip › Agreement Transcripts/EAR_Howler_I (agreement statements responses).docx]

Speaker 2: I think strongly disagree.

Speaker 2: Well, I'd say that in order for us to be able to have programs about the Del Toro it's necessary for us to be here. I think that's the main reason.

Speaker 2: I would say I agree because I think Bocas Del Toro is a really good place to have programs, but I'd say we definitely have had some challenges, which is why I don't want to stay strongly agree, such as the remoteness of having students here. That was the main challenge with this last group, is making sure they don't go island crazy, especially because they're from the big twin cities in Minnesota.

Sorry about that, I don't know what they're doing. That's why I'd say agree because we have had some challenges.

Speaker 2: I feel like probably the school field studies and ST ...

Speaker 2: Yeah, Smithsonian as well as ... What's the other one? School of Conservation ... SCRI. School of Conservation Research something. Tropical ... STRI. School of Tropical Research in ... Yeah.

Speaker 2: I don't remember the exact acronym for them. They're different in the sense that they offer a program to people and then the people decide whether or not the program fits, whereas we offer our services to people and then we make the program fit to people, if that makes sense. That's what makes us different from those other organizations and they might have other advantages and disadvantages than we do.

They also have been around a lot longer than we have.

Speaker 2: They're more like, "This is what we're going to be studying if you want to join us," whereas we're like, "What do you want to study? We'll try to figure it out."

Speaker 2: I'd say agree. I'd say ours, at the moment, since we're still starting out, probably have more of an effect on the students versus the community at this moment. Since this is only my first programs that I'm running, I'm trying to get it to be more involved in the community and that's what the University wants. It's just difficult here sometimes to get the trust with the community to be able to do stuff.

I'd say at the moment, it's more the students that have an impact, or they feel more impacted than I'd say the impact ... I wouldn't say we have an insane, amazing impact with the community, but I do know that a lot of our students after they left us last summer, were like, "This is really ..." Especially the ones that were Floating Doctors, Smithsonian, stuff like that, they were like, "This center really had a big impact on me."

I'd say at the moment, it's more that we have the impact on people that do the program versus the community, but we're working on having more of a presence here and everything.

Speaker 2: I think that kind of doesn't really fit yet just because we have only been ... I've only been here for about a year now and I feel like we haven't really been around long enough to be able to say ... I don't know. I feel like I can't really give you a for or against.
