# Supplementary material for: Is there no “I” in team? Potential bias in key informant interviews when asking individuals to represent a collective perspective
Source: PLoS One. 2022 Jan 14;17(1):e0261452. doi: 10.1371/journal.pone.0261452 (PMC8759660; doi:10.1371/journal.pone.0261452)
Supplement: S2 File — This zip file contains the original transcriptions of the interviews used in for this study. (ZIP) [file pone.0261452.s002.zip › Agreement Transcripts/CBT_Urchin_I(agreement statements responses).docx]

Speaker 3: Yeah.

Speaker 3: No, it's okay.

Speaker 3: Depends on the context that you view it in.

Speaker 3: So, I don't know.

I disagree, I guess.

Speaker 3: I'm agree.

Yeah?

Speaker 3: In doing this job? In doing the chamber work or?

Speaker 3: Yeah.

Speaker 3: Yeah. Because, once again, like I said, all of this different aspects have to be involved to be able to be sustainable. Sustainable in the tourists, it's the biggest thing. If it's not sustainable, it's not gonna be friendly, it's not gonna be friendly, you're not gonna be here, if you're not gonna be here, it's a ...

So yeah, I'm pretty sure a lot of different organizations they want to be, participation of community to be doing the job.

Speaker 3: Making sustainable.

Speaker 3: Oh, I'm 100% agree.

Speaker 3: Yeah.

Speaker 3: Strongly agree.

Speaker 3: For what?

Speaker 3: I'm agree.

Speaker 3: It's in the, I don't know if you ever see how the graphics of how the develop a country or how develop different places. So you normally are in a curve learning, you gotta have the big figure, you don't show up in any map, nobody sees you, nobody knows you. If you got one customer, it's 100% profit right now, because you have enough customers. Where it gets in the point is, when you have a thousand customers, it gets in the point is that, 'Okay, we really need to focus and make it better.' The way to make it better is to do more work.

Speaker 3: So, it's more important now than it used to be back 10 years because back 10 years it used to be a lot more easy. It was less business, with less people to try to claim. I mean, human nature is selfish. We all thinking ... 'Blah, blah, blah!' Earlier with the people with the garbage, the guy who's doing the impact stories, I tell him, it's very funny because, when you do an impact study, the idea of making an impact study is to figure out why the problems that you can come along all the situations to be able to minimize the problems. And, in that way, make it sustainable, making sure you don't destroy everything around and you don't kill like two thousand fish, which is, maybe like one or two because we are touching something that has to be ... You know, what I mean?

And he tells me, 'You're the first person that I make more than 300 of those, and you're the first person that ever tell me, as a commerce business and a business owner, that it's safe.' And he want to know what he can do to prevent those situations.

Most of the people, I think, 'Ah, I gonna let it, I gonna make it happen but I don't think about consequences.' If you thinking like that and most people thinking like that. Obviously, you need to figure out how to put the rules.

So then, somebody came and he's like, 'Well, I gonna dump my diesel into the ocean every day because I don't care. It's not gonna make a difference. It's not unique.' When you have a hundred then. When you have the rules set, you don't have a fish problem.

And it's not gonna be a sustaining one, and you're not gonna have tourism. And all that list that you have in there, is come up. It has to be a lot more aware of what's going on and how your things affects somebody else. People don't [inaudible 00:46:01]. And nobody thinking like if they have to leave the school, you have to leave your family, your girlfriend, your wife, whatever, to do this part of your program to be finished with school. Only you know it. None of the other people know and be able to acknowledge the issues too.

I don't know if I answered that question.
