# Supplementary material for: Is there no “I” in team? Potential bias in key informant interviews when asking individuals to represent a collective perspective
Source: PLoS One. 2022 Jan 14;17(1):e0261452. doi: 10.1371/journal.pone.0261452 (PMC8759660; doi:10.1371/journal.pone.0261452)
Supplement: S2 File — This zip file contains the original transcriptions of the interviews used in for this study. (ZIP) [file pone.0261452.s002.zip › Agreement Transcripts/CBT_Hammerhead_Translation(agreement statements responses).docx]

**Reinaldo:** Right here, we would not really go to another that we work. Because this **[unintelligible 00:12:14]** is very lot of p oakm. But the people so far out, do need the job here and **[unintelligible 00:12:21]** . "Stranger, how are you? **[Unintelligible]** **00:12:24]** . "We lose a lot of clients to that job. We go out to do one, it's not really an agreement for nothing.

**Reinaldo:** Yes. What you do not like to do, you do not do. Too much stranger taken all the tourists there. Then, it's a lot of money and we do not have that money. Everything package and just leave it about.

**Reinaldo:** Yes, sure.

**Reinaldo:** Yes, really. Just only many people **[unintelligible 00:13:16]** Belize, any experience, so nothing like really when they came here.

**Reinaldo:** No, Amael is good. What I'm telling you is that many people **[unintelligible 00:13:27]** . In Belize, you **[unintelligible 00:13:29]** it's nothing like here. I have to believe what I'm telling you.

**Reinaldo:** Everyone has a different answer. Sure, it's likely they can agree. Normally, everyone has a different answer.

**Reinaldo:** Our work in Bocas. Really Yes, we need a lot of work.

**Reinaldo:** Very necessary.

**Reinaldo:** Yes.

**Reinaldo:** Yes.

**Reinaldo:** Yes, because every time there is more people and it has to be more important. More people coming in, so it has to be a very important work.

different than we in the place because they have everything that owner of the association **[unintelligible 00:17:34]** .

We not, we just forward it. **[unintelligible 00:17:37].** It's different at them. They have different rules, that's what I said.
