# Supplementary material for: Is there no “I” in team? Potential bias in key informant interviews when asking individuals to represent a collective perspective
Source: PLoS One. 2022 Jan 14;17(1):e0261452. doi: 10.1371/journal.pone.0261452 (PMC8759660; doi:10.1371/journal.pone.0261452)
Supplement: S2 File — This zip file contains the original transcriptions of the interviews used in for this study. (ZIP) [file pone.0261452.s002.zip › Agreement Transcripts/GOV_Shrimp_Translation(agreement statements responses).docx]

**Interviewee:** Of course I do.

**Interviewee:** Strongly agree.

**Interviewee:** Why do you agree? Because if Bocas del Toro is just a province of a country, which has 10 provinces, and we have a fairly small staff and a fairly small team, and we can do it. All provinces have a similar team, and much larger with much more staff than us. So what is done here, I imagine that is also being done in the other provinces. All applied to the sector, no sector is equal to the other.

Because you can see, for example, we have here a Caribbean that covers what is the area of ​​Bocas del Toro, and Columbus has the same Caribbean and totally different. So I imagine that Colon's staff should be doing the same, and if it is not the same it is very similar. But yes, of course .

**Interviewee:** Yes, very much in agreement.

**Interviewee:** Maybe not, because I feel that Bocas del Toro does have a sea, it has an island, it has a mountain, it has nature, it has agriculture and it has tourism. It has common users and has a company, it has artisanal fishing, thank God we do not have the industrial and the tr down that we are doing in the area, we do it applied to what we have.

Suddenly in the Chiriquí area, they will think that the most appropriate is theirs. The Colon area will say that the best part to work for is Colon, and the one in Panama West will say the same thing . Everyone in the area. They can not say that Bocas del Toro is the best, but it is a place that has everything that is needed to work and develop the community, organizations and institutions have everything that is needed to apply the four directions that They are the ARAP.

**Interviewee:** Because you tell me that if I think that Boca del Toro is the best place to develop.

**Interviewee:** For me yes, strongly agree, but if you interview **[Unintelligible 00:30:33]** each one is regional and defends its region. For me it is the province that has the four activities most applicable to what is in itself the ARAP

**Interviewee:** Claro.

**Interviewee:** Strongly agree.

**Interviewee:** I feel that there should be more institutions, or the same institutions that already exist had a department that would give better attention to the sector that is not accessible by road. Everyone caters where the cat arrives, but they have neglected the coastal marine sector, even the insular sector, they have neglected it in an incredible way.

We have had the responsibility or the need to act as housing, education, health, so many things that suddenly if you analyze it we are not the ones who have competence.

**Interviewee:** Yes and no. Yes, because the nature of the human being implies that if dad is not there, he will do what he wants, and not because we have trained the communities so much that everything has already been made available to us. Before for a person to understand that something was wrong, it was a problem. They already know that they do not, and if they do and they arrive at the place they know they are guilty and they look down and even say, "Yes, excuse me, let's see how I try to solve what I did wrong".

When we want to start a project in a community, it used to cost us a lot, we had to take a person or a family in a community and forget about the rest, and try to make everything work for others to observe and everyone would like to be Well, like him, the projects were irrigated. Not now, now we come, "We want to make a shrimp project," "I want," everyone wants.

Everyone now believes in the institution, believes in projects, believes in conservation, people have changed their mentality, that's why I say yes and no.

**Interviewee:** Yes nothing extreme because-

**Interviewee:** I feel that if tomorrow the institution disappears or the government decides not to have an ARAP branch in Boca del Toro anymore, an address in Boca del Toro, the projects will not disappear, they will remain in the weather. What if maybe I could increase that again I would have to be present, be it capture or illegal fishing due to lack of work places.

To people by necessity it can not be that I arrive at the house today in the afternoon and my girl tells me that she needs a job from the school and she wants $ 10 and I do not have it , that hard. Or that I arrived at night and they have cut off the electric power because I could not pay it, because the economy does not give. I go out to sea and when I come in, if I did not catch the fish I wanted, I come picking up snail, lobster, turtle all there is, just to bring the economy, the income to the house.

That's something that has, I say put your hand in the chest the central government. Instead of giving subsidies and giving things that were suddenly done with a good idea, a good initiative, but in the end it is not the right thing to do, it is to make alternative projects sothat people feel that everything has a cost, has a value and We must take care of it. Things can not be for free and there is **[unintelligible 00:36:21]** .

**Interviewee:** Or institutions. That question is a little compromising. Imagine if I said no and the others say, "Wow, what happened to this **[unintelligible]** ? What happens here is that we need them to join in order to be given the attention that the population deserves, but no other institution directly applies to everything that was spoken.

We have the Ampyme, the Ampyme gives you a training, it gives you a certificate of training and in that it gives you a letter that lets you understand that you can compete up to $ 1,000 for a project of a micro project and before two, three years if everything is applied and your project is presented to you.

Armas a project with Ampyme, but Ampyme has more to do with nothing else, you go to INADE and INADE teaches what you believe in the community you need and if today you say that outboard mechanics, 40 people are trained, if there are 40 trained outboard mechanics, how are you going to make profitable if you are going to dedicate to that if there are 40 more involved with you, you can only fix your engine, you are understanding me.

You go then to the MIDES, the MIDES looks for the disabled, to my guardian and donates but they are donations, they are subsidies that are going to give you, for what time? When you start to give it, no matter how little something is to someone for more than 10 years, you make a charge for the State, you make a person who will live the whole life of the subsidy and who will not want to work. You are creating a burden instead of doing it the way it should be because you had to make a donation or a subsidy, but with certain responsibility.

I am going to review you to the year and to the year you are going to me to say that you did. After a year you will tell me if you got a job, after a year you will tell me that you should give us this recourse to ARAP and ARAP does a project there and instead of giving you $ 100 every month, I'll give you a project that you have to market or work to earn the $ 100.

I feel it could be but it is not happening, MIDAS is very well spoken, the MIDA S is so large and so extensive, it governs us as a Ministry, we are part of the MIDAS, but the MIDAS is very extensive, very broad, encompassing all and I do not see results. When you see that in Panama there is a good genetics of cattle, it is not MIDAS, it is from a millionaire who brought it and when you see you see them present there you see beautiful things, but none of them is from my MIDAS.

I do not want him to misinterpret me either, but it's what I see, I see that the **[unintelligible 00:39:48]**is up to here starts to see what we're going to do with the **[unintelligible 00:39:52]**but not at all you can plant the **[unintelligible] 00:39:53**and you can plant a **[unintelligible 00:39:55]**and when you go to harvest it in five years, and people died of hunger there are things that can be left and things No, he brings me a good cocoa product, hybrid, sows it, we arrange it with the MIDAS, the research brings us a disease, a pod and everything that we had that was native died, that it was natural that we produced. They bring us a variety of rice. Before we had a small one that did not fall, now you brought us a smaller one that the duck eats or a very big one that the wind knocks down. At the end of the story, if we are seeing it from a point of view, we are doing it from a commercial point of view.

Leave it out. We are already seeing. I believe that the only institution that does what it should in Panama is a passport, which gives a passport for people to travel. Do you understand what I said?

If we go to the Ministry of Labor, there are people with work permits, who come from another country and have a work permit, but do something else. If we go to Migration, the people who enter, come to do something but it is something else.

In transit, you see that it has to do with a regulation, a cos , but you always find the cars. As a Panamanian, I believe that everything is done as it should be, but it does not apply as it should be correct.

All institutions have their mission and vision, but it does not develop. It hurts, but it is the truth. I would like so the MIDAS, because if I have a community that has a project, I need a bank that finances with a low interest. The BDA assists us, supports us, accompanies us. We fill communities with health, which saddens us. That he measures with his social work .

That the MIMI for housing, for families with limited resources, but it is not like that. We will believe that, with decentralization and local governments now, it will change, but that is up to the people. The people choose what they deserve, they get what they deserve because they choose it, but I still do not see much light at the end of the tunnel.
