# Supplementary material for: Is there no “I” in team? Potential bias in key informant interviews when asking individuals to represent a collective perspective
Source: PLoS One. 2022 Jan 14;17(1):e0261452. doi: 10.1371/journal.pone.0261452 (PMC8759660; doi:10.1371/journal.pone.0261452)
Supplement: S2 File — This zip file contains the original transcriptions of the interviews used in for this study. (ZIP) [file pone.0261452.s002.zip › Agreement Transcripts/CBT_Sloth1_Translation((agreement statements responses).docx]

**Interviewee:** No.

**Interviewee:** I think not. It's community, it must be there.

**Interviewee:** It could be, yes.

**Interviewee:** Agree.

**Kelly:** Is there other organizations can you answer the same? Is there other organizations--?

**Interviewee:** Maybe the 10 communities would say the same [laughs].

**Interviewee:** Yes, because I think it should be, I do not know if that's what it means, but we have been trying to have an office in Bocas but there is no place, there is no place to receive clients or visitors who send us directly from the communities. But we do not have any of that in Bocas.

**Interviewee:** Island.

**Interviewee:** No, because at first yes, because there were fewer projects on different islands, now, there is a lot of competition and that is why few of us are here.

**Interviewee:** We did not do workshops before, but always with the initiative that we seek and with the support of **[unintelligible 00:36:04]** , it is an association, an organization that works with the children of the school that is here as well and with them we have built a small school for the kindergarten and the teacher's table.

**Interviewee:** With them we have had that opportunity, they have brought us clients to do this type of workshops and that we have been doing for two years here.
