# Supplementary material for: Is there no “I” in team? Potential bias in key informant interviews when asking individuals to represent a collective perspective
Source: PLoS One. 2022 Jan 14;17(1):e0261452. doi: 10.1371/journal.pone.0261452 (PMC8759660; doi:10.1371/journal.pone.0261452)
Supplement: S2 File — This zip file contains the original transcriptions of the interviews used in for this study. (ZIP) [file pone.0261452.s002.zip › Agreement Transcripts/CBT_Bat_Translation (agreement statements responses).docx]

**Interviewee:** You were from Bocas?

**Interviewee:** Outside Isla Colón?

**Interviewee:** I think so.

**Interviewee:** Strongly agree.

**Interviewee:** Yes, I think so.

**Interviewee:** Strongly agree.

**Interviewee:** It's like that. Regarding that question, it is also a yes.

**Interviewee:** Yes. Strongly agree. Totally agree.

**Interviewee:** Yes

**Interviewee:** Agree.

**Interviewee:** Strongly agree.

**Erick:** Right. We are the largest group, because here in Panama [sic] we have eight indigenous types. There is the Kunas, there is the Teribe, there are the Emberá, San Blas, we are, who are Ngäbe. We are the indigenous with more population, in territory and in population. That's why I say yes, I totally agree.
