# Supplementary material for: Is there no “I” in team? Potential bias in key informant interviews when asking individuals to represent a collective perspective
Source: PLoS One. 2022 Jan 14;17(1):e0261452. doi: 10.1371/journal.pone.0261452 (PMC8759660; doi:10.1371/journal.pone.0261452)
Supplement: S2 File — This zip file contains the original transcriptions of the interviews used in for this study. (ZIP) [file pone.0261452.s002.zip › Agreement Transcripts/CBT_Lobster_Translation(agreement statements responses).docx]

**Gilberto:** Disagree, we are doing here a lot.

**Gilberto:** Okay.

**Interviewer 2:** Agreement.

**Interviewer:** [laughs] "Do you have other organizations that do the same thing that you do".

**Gilbert** **or:** Yes.

**Interviewer:** Which ones do you think of the same thing that you do?

**Gilberto:** What does he say?

**Interviewer 2:** The question?

**Gilberto:** No.

**Interviewer 2:** No, why?

**Gilberto:** It depends too. Because if there are other organizations that fit with us. An agreement that is for **[unintelligible 00:17:56]** .

**Interviewer 2:** Depends.

**Gilberto:** Agreement.

**Gilberto:** Very much.

**Interviewer 2:** Very much, very much in agreement.
