# Supplementary material for: Is there no “I” in team? Potential bias in key informant interviews when asking individuals to represent a collective perspective
Source: PLoS One. 2022 Jan 14;17(1):e0261452. doi: 10.1371/journal.pone.0261452 (PMC8759660; doi:10.1371/journal.pone.0261452)
Supplement: S2 File — This zip file contains the original transcriptions of the interviews used in for this study. (ZIP) [file pone.0261452.s002.zip › Agreement Transcripts/CBT_Hawk_Translation (agreement statements responses).docx]

**Interviewee 2:** The first, right? It says, "You can do the work outside of Boca."

**Interviewee 2:** Out of Boca?

**Interviewer 2:** If the work you do can be done outside Boca.

**Interviewee 2:** In another province, can it be too?

**Interviewee 2:** I do not think so.

**Interviewer 2:** Disagree or strongly disagree?

**Interviewee 2:** Disagree.

**Interviewee 2:** Yes.

**Interviewee 2:** Strongly agree.

**Interviewee 2:** I think so, because all the organizations here on the island-- we all want-- I say yes because everyone is always in tourism, what is Boca del Toro offer as a tourist destination, we all always have the same idea .

**Interviewer 2:** They are connected. The same-

**Interviewee 2:** Strongly agree.

**Interviewer 1:** Over time, the importance of Bocas for our work has increased.

**Interviewee 2:** Yes, the work has increased a lot.
