# Supplementary material for: Is there no “I” in team? Potential bias in key informant interviews when asking individuals to represent a collective perspective
Source: PLoS One. 2022 Jan 14;17(1):e0261452. doi: 10.1371/journal.pone.0261452 (PMC8759660; doi:10.1371/journal.pone.0261452)
Supplement: S2 File — This zip file contains the original transcriptions of the interviews used in for this study. (ZIP) [file pone.0261452.s002.zip › Agreement Transcripts/BUS_Dolphin_Translation ((agreement statements responses).docx]

**Interviewee:** No. Only in Bocas del Toro.

**Interviewee:** Strongly disagree, disagree, agree, strongly agree. Number three

**Interviewee:** Yes, I think so.

**Interviewee:** Number three.

**Interviewee:** Because we live here, family here, everything here. If you went to other place, it's tourist but you have to begin again. Here we have family, friends, everybody knows you and it's more easy to work in the area, but if you went to a different place you have to start again, make friends, make people you know. That is difficult.

**Interviewee:** I do not see nobody more do that.

**Interviewee:** Yes, there's only organizations tourist. Everybody by himself, everybody apart. They have their own politics, we have our own politics, everybody do the same politics.

**Interviewee:** Yes, the number three.

**Interviewee:** Yes. It is necessary. We have problems everyday here. Always you have to work. That is the thing here. Everybody works here every day. We have a lot of people here in town when it's the end of the year, in the 25, 26, 27 in Christmas, in December, eat a lot of people in town. Everybody have work. When it is the end of the year, this pod is filled here in Boca del Toro. Full.

**Interviewee:** Yes

**Interviewee:** Because when we start the tourist, only the people go to the one island. People do not know about other different things. We have beautiful natural places we have in the area. Right now the people explore it and the more pass the year you see more activity in town. A lot of tourists like to come here because this is in Caribbean side, we have a lot of competition, in Cuba, Puerto Rico, Dominican Republic, the Bahamas. All, Caribbean side we have a lot of place with white sand like here, but we try to fight it for this place has the more best security, this place has the more good service for the tourist, and the tourists go with the good expression of the island. That is we want.
