# Supplementary material for: Is there no “I” in team? Potential bias in key informant interviews when asking individuals to represent a collective perspective
Source: PLoS One. 2022 Jan 14;17(1):e0261452. doi: 10.1371/journal.pone.0261452 (PMC8759660; doi:10.1371/journal.pone.0261452)
Supplement: S2 File — This zip file contains the original transcriptions of the interviews used in for this study. (ZIP) [file pone.0261452.s002.zip › Agreement Transcripts/EAR_Turtle_I (agreement statements responses).docx]

Pete: Okay. All right.

Pete: Yes, we could.

Pete: Let me see. Let me think about this for a second.

Pete: I agree.

Pete: I agree. We could. But yeah, let me put a little disclaimer in there. The part of the reason we chose this area was the reasons I had mentioned earlier. The fact that we have the forest and the marine ecosystems right next to each other.

The one thing I didn't mention, I didn't think it was important at the time, but now I see that it is, was the cost of doing it here was extremely low. And we're not, as I said, Stry or other organizations that have a lot of resources.

And so we had to choose a place that was economically feasible for us to do, and this was it. 30 years ago to 25 years ago, 25 years ago, It would have been, you wouldn't believe how inexpensive it was here.

Pete: You could buy an acre of land for 60 dollars. You could buy a waterfront house for 2,000 dollars when I first got here 23 years ago..

Pete: Yeah. And we had, at that time at the old location, a woman cooking for us. In fact, she runs the Yaddish Morie Restaurant. I don't know if you stopped by there when you came out. Did you take the water taxis around or did you walk out to the beach, Starfish Beach?

Pete: Okay. You walked right by the restaurant there on the left right where the bus is. Where the bus is.

Pete: The woman who cooked for you was our cook for the first six or seven years that we were here. Her name is Juani. And she could cook three meals a day per person for six dollars and 50 cents.

Pete: And make a profit doing that. That's how inexpensive it was. So yes, we could do this almost anywhere where there is tropical reefs and rainforests. Could be Costa Rica, although they don't have much in the way of coral reefs.

But you certainly could do it in places like Serenem or Venezuela, Columbia. Not so much in the Lesser Antilles because those are dry forests for the most part. Although the further south you go toward Trinidad, they become wet forests. So there's lots of places we could do it.

Pete: Okay? But so I agree with an explanation.

Pete: Mm-hmm (affirmative)-

Pete: I agree.

Pete: I agree with that right now. Yeah. If you would have asked me this 10 years ago, I would have said I strongly agree.

Pete: But things are changing very rapidly. Things have, Panama has now become one of the most expensive countries in Latin America. And so that has hurt us considerably. For that very same reason that we came here in the first place was the fact that it was so inexpensive. Now it's very, very expensive. The cost of living here is higher than it is in Gainesville, Florida where I live normally. Yeah.

Pete: Oh good question. Well the only other organizations that I'm aware of that are similar to ours, well there are several actually. There's The School for Field Studies. And they're here primarily because, well for several different reasons.

They're here because they wanted a venue that they had both forest and reef too. And, but a place that was also developing tourism. So their primary focus is resource, environmental science and resource management and that kind of thing. Kind of resource conservation in tourism. That's the sort of thing they're doing now.

And so they chose this spot, I think because they were in Mexico and they had to pull out of Mexico. And so now they're down here. I don't know if they would consider this the only place they could do this, but they do like being here. And they're currently here to a very small degree because we were here first and we, I know their academic dean very well. I know Leonore very well, I know Cinda very well.

And so when their academic dean, Mark, decided to, he got his PhD working here. And so when he started his position with School for Field Studies, it was only a natural idea for them to come down here. At one time, they were considering using the field station here.

Pete: Yeah. But they chose to rent a hotel instead in town. Which I think was the better, the smarter move on their part. For the kind of things that they do.

Pete: Well there's one other, I'm sorry.

Pete: I just wanted to mention there's also Stry of course. And then, but there's a University oF Minnesota also has a location here over in Red Frog. And I met with their liaison there between the Red Frog, I don't know what it is, I've never been there to their station or center or whatever it is they have, and the University of Minnesota.

We had a conference the other day and talked about potentially working together and ways that we might. And so that's another one. But those are the only ones that I know of that are academically based. Yeah.

Pete: For Bocas Del Toro?

Pete: Not as much. I would say, what was all the categories again?

Pete: I'm gonna say disagree on that one.

Pete: And the reason is we try to make an impact on this community. And by example and by doing various kinds of projects. We don't seem to be as connected to the community in town as others are. And that's partly due to the fact that we're kind of remote here. And so I don't know that our being here really helps the community all that much in town. We do help the local community, the school. But in town, maybe not so much.

You know, we've done a number of different programs in town early on. We tried to introduce cloth bags 20 years ago. And now you have, they just got rid of the plastic bag use in town. And that was done by a consortium of different people and we weren't involved in that.

And I think that most of the people who have come in the last 10 years or so aren't even aware that we exist. Yeah. So you wouldn't be here at all if it wasn't for the people at the School for Field Studies, right?

Pete: So I would say I disagree with that. Yeah.

Pete: Again, I would have to disagree with that as well. I think the work that we do here, Bocas itself as a community, as I said, it doesn't really benefit that much from our being here, other than perhaps financially. Because that's where we buy everything, right? And so we do spend a fair amount of money here every year.

But our main purpose is to educate people so that they can make better choices, environmental choices in the future, or so that they can go on to advanced degrees. So our main interest is education, whoever and wherever they come from. And if they can use that education and their knowledge to plan better for the resource management of Bocas Del Toro, then that's great.

Some people have decided to reside here and are actually working in that area. Lanor, for example, was a student of ours. She originally worked in our sea turtle program about 20 years ago, 18 years ago. And then also did community education for three years with us. And so what she did did benefit the local community to some extent. But sadly it doesn't seem to stick. It really doesn't.

One time we did a project where we cleaned up Sandfly Beach. I don't know if you know where that is. That's the beach in the Fairground area. Where that new park is and the house I was telling you about. You can see off to the left and the surface coming in. Yeah, just before you get to the Saigon area.

Anyway, we decided to do a beach cleaning. And this was before Smithsonian came in or anybody else or School for Field Studies or anything like that. And what happened was, we went out there on the beach and we attracted kids to come out and clean up the beach. And then there was gonna be a party with hot dogs and stuff after that.

And they did. They all came out. We were working with the local schools and they all came out and they cleaned up the beach. And during the little party we had afterwards, there was a little boy and he was finishing his Kool-aid or whatever it was he was drinking. And as soon as he got done with it, just threw it right on the ground.

And like he didn't really make the connection between what we were doing and why we were doing it. But to her credit, an older girl sitting next to him starts yelling at him. "What did you do that for? We just got done cleaning the beach." So maybe it helped a little bit.

But you've seen what Bocas looks like. People have put up trash cans all over the place. It's just hard to change a culture. It really is. And sometimes I wonder whether that's our responsibility or even our obligation to do so. I mean it's their culture, right? But in this case it's one in which also affects the environment. And I think that's why it does need to be improved on some level. But I guess that's it.
